# Increased use of total shoulder arthroplasty for osteoarthritis and improved patient-reported outcome in Denmark, 2006–2015: a nationwide cohort study from the Danish Shoulder Arthroplasty Registry

**DOI:** 10.1080/17453674.2019.1633759

**Published:** 2019-06-26

**Authors:** Jeppe V Rasmussen, Alexander Amundsen, Anne Kathrine B Sørensen, Tobias W Klausen, John Jakobsen, Steen L Jensen, Bo S Olsen

**Affiliations:** a Department of Orthopaedic Surgery, Herlev Hospital, Herlev;; b Department of Clinical Medicine, University of Copenhagen;; c Department of Hematology, Herlev Hospital, Herlev;; d Department of Orthopaedic Surgery, Aalborg University Hospital, Aalborg;; e Department of Clinical Medicine, Aalborg University, Denmark

## Abstract

Background and purpose — Osteoarthritis has become the most common indication for shoulder arthroplasty in Denmark, and the treatment strategies have changed towards the use of anatomical total shoulder arthroplasty and reverse shoulder arthroplasty. We investigated whether changes in the use of arthroplasty types have changed the overall patient-reported outcome from 2006 to 2015.

Patients and methods — We included 2,867 shoulder arthroplasties performed for osteoarthritis between 2006 and 2015 and reported to the Danish Shoulder Arthroplasty Registry. The Western Ontario Osteoarthritis of the Shoulder (WOOS) index at 1 year was used as patient-reported outcome. The raw score was converted to a percentage of a maximum score. General linear models were used to analyze differences in WOOS.

Results — The proportion of anatomical total shoulder arthroplasty and reverse shoulder arthroplasty increased from 3% and 7% in 2006 to 53% and 27% in 2015. The mean WOOS score was 70 (SD 26) after resurfacing hemiarthroplasties (n = 1,258), 68 (SD 26) after stemmed hemiarthroplasty (n = 500), 82 (SD 23) after anatomical total shoulder arthroplasties (n = 815), and 74 (SD 23) after reverse shoulder arthroplasties (n = 213). During the study period, the overall WOOS score increased with 18 (95% CI 12–22) in the univariate model and 10 (CI 5–15) in the multivariable model, and the WOOS scores for anatomical total shoulder arthroplasty increased by 14 (CI 5–23).

Interpretation — We found an increased WOOS score from 2006 to 2015, which was primarily related to a higher proportion of anatomical total shoulder arthroplasty and reverse shoulder arthroplasty towards the end of the study period, and to improved outcome of anatomical total shoulder arthroplasty.

The Danish Shoulder Arthroplasty Registry (DSR) was started in 2004 (Rasmussen et al. 2012). Each year a report is published, which includes results and recommendations. Furthermore, peer-review publications from the registry are described in the annual reports and the results are presented at annual meetings of the Danish Society of Shoulder and Elbow Surgery and at the Danish Society of Orthopedic Surgery. Because of limited information from randomized trials and large observational studies, data from the DSR are an important source of information regarding patient-reported outcome, arthroplasty survival rates, and reasons for revision of different arthroplasty types.

Osteoarthritis has become the most common indication for shoulder arthroplasty in Denmark. Furthermore, the treatment strategies have changed towards the use of anatomical total shoulder arthroplasty and reverse shoulder arthroplasty. We hypothesized that changes in the use of arthroplasty types have improved the overall patient-reported outcome of shoulder arthroplasty for osteoarthritis.

We studied changes in the use of arthroplasty types for osteoarthritis in Denmark from 2006 to 2015, the patient-reported outcome of different arthroplasty types, and whether changes in the use of arthroplasty types have changed the overall patient-reported outcome from 2006 to 2015. 

## Patient and methods

### Source of data

Data were obtained from the DSR. All Danish hospitals and private clinics report patient and surgical data at the time of operation. Every year the completeness of reporting is calculated by comparing data from the DSR with data from the National Patient Register—an administrative database used by the Danish healthcare authorities to reimburse expenses for any hospital treatment including shoulder arthroplasty. In its first 2 years of existence, the completeness of reporting to the registry was low, and therefore 2004–2005 is now regarded as a trial period, and the results usually not included in research. Reporting to the registry became mandatory in 2006. Since 2007, the completeness has been above 90% and the completeness for this report was 93% for both primary and revision arthroplasties. A patient-specific identification number given to all Danish citizens at the time of birth or immigration was used by the registry to link a revision to the primary procedure. The identification number was also used when information concerning death or emigration was obtained from the National Registry of Persons.

### Inclusion criteria

We included all shoulder arthroplasties performed for osteoarthritis between 2006 and 2015 that had been reported to the DSR. In that period it was possible to report more than 1 indication, so for this study we used a hierarchy including the following indications ranked in descending order: acute fracture; fracture sequelae (nonunion; malunion; previous osteosynthesis; fractures reported together with osteoarthritis or humeral head necrosis); inflammatory arthritis; rotator cuff arthropathy; and osteoarthritis. If more than 1 indication was reported only that with the highest rank in the hierarchy was recorded.

### Patient-reported outcome

The Western Ontario Osteoarthritis of the Shoulder (WOOS) index was not assessed preoperatively but assessed by a postal survey at 1 year. For economic and logistical reasons, the survey was only sent to the patients once. In case of revision, death, or emigration within the first year, the WOOS score cannot be obtained.

WOOS is a disease-specific questionnaire that measures the quality of life of patients with glenohumeral osteoarthritis (Lo et al. 2001). The total score ranges from 0 to 1,900, with 1,900 being the worst. For simplicity of presentation, the total score is converted to a percentage of the maximum score, with 100 being the best. The Danish version of WOOS has been translated and cross-culturally adapted (Rasmussen et al. 2013). The minimal clinically important difference on the WOOS has never been validated. For the present study we used 190 (i.e. 10% of a maximum score), which was extrapolated from studies validating the minimal clinically important difference of other shoulder-specific outcome measures.

### Statistics

WOOS scores were described using mean value and SD. A general linear model was used to analyze WOOS scores. Age (< 55 years, 55–75 years, > 75 years), sex, arthroplasty type, and year of surgery were included in the analyses. Interaction between year of surgery and arthroplasty type was used to study change during the studied period for each arthroplasty type. Estimates were given with 95% confidence intervals (CI). Due to the large sample size we considered it appropriate to use a parametric model. Linearity of year of surgery was investigated using a smoothing spline function. Patients with bilateral arthroplasty (n = 249) were included in the analysis as if the arthroplasties were independent. The analyses were performed using SPSS version 22.0 (IBM SPSS Statistics for Windows, Version 22.0. IBM Corp, Armonk, NY, USA). The level of significance was set at p < 0.05 and all p-values were 2-tailed.

### Ethics, funding, and potential conflicts of interest

According to the regulations in Denmark, this study did not need permission from the National Committee on Health Research Ethics. No funding was received. There were no conflicts of interest to be declared related to this study.  

## Results

### Study population

2,867 primary arthroplasties for osteoarthritis were reported to the registry during the study period. There were 1,732 (60%) women. Mean age was 67 (SD 10) years. 13% patients were aged 55 years or younger, 65% patients were between 56 and 74 years, and 22% patients were 75 years or older. There were 1,258 resurfacing hemiarthroplasties, 500 stemmed hemiarthroplasties, 815 anatomical total shoulder arthroplasties, and 213 reverse shoulder arthroplasties. 68 arthroplasties were recorded as “others,” which included 21 stemless hemiarthroplasties and 47 stemless total shoulder arthroplasties. 13 cases were recorded with a missing arthroplasty type (Table 1). 249 patients had bilateral arthroplasties.

**Table 1.** Anatomical total shoulder arthroplasty (TSA), resurfacing hemiarthroplasty (RHA), stemmed hemiarthroplasty (SHA), and reverse shoulder arthroplasty (RSA), number (%)

	TSA	RHA	SHA	RSA
Sex				
Women	543 (32)	691 (41)	297 (18)	156 (9)
Men	271 (25)	567 (52)	203 (18)	57 (5)
Age				
≥≥ 75	175 (29)	194 (32)	132 (22)	102 (17)
56–74	590 (33)	811 (45)	306 (17)	107 (6)
≤≤ 55	50 (14)	253 (69)	62 (17)	4 (1)

The table does not include arthroplasties that were recorded as others (n = 68) or with a missing arthroplasty type (n = 13).

### Distribution of sex, age groups, and arthroplasty types over time

**Figure 1.**
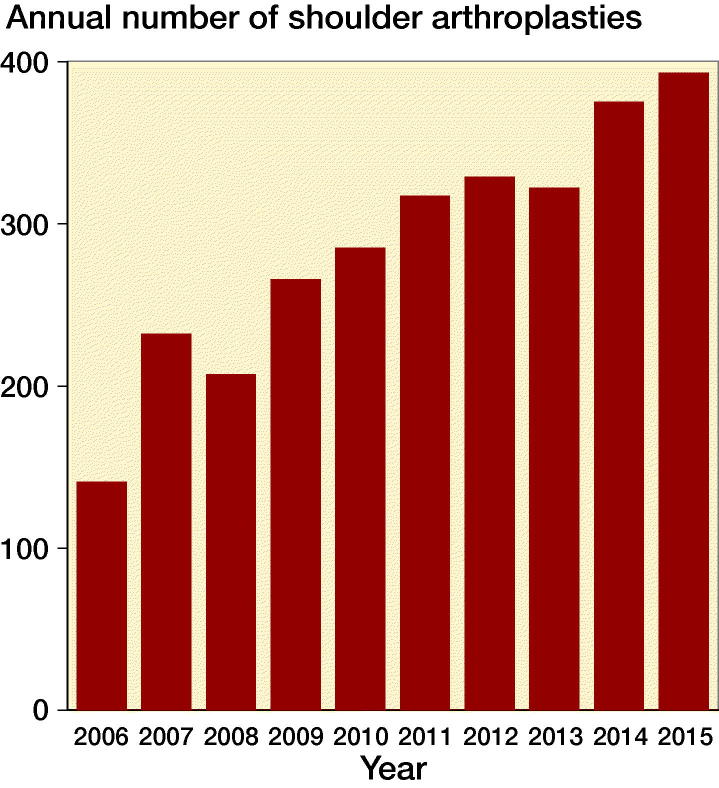
Annual number of shoulder arthroplasties for osteoarthritis from 2006 to 2015.

**Figure 2.**
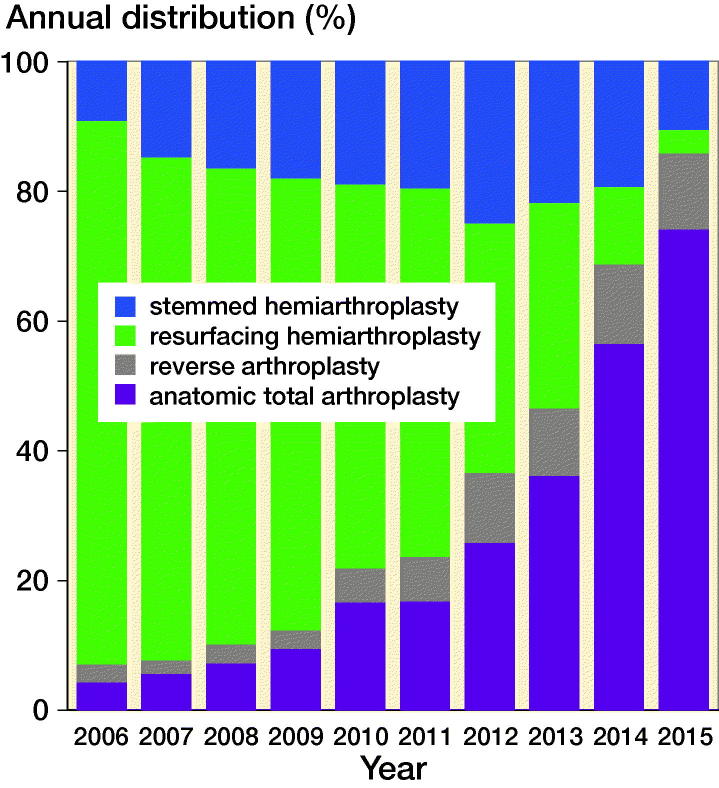
The proportion of stemmed hemiarthroplasty (blue), resurfacing hemiarthroplasty (green), anatomical total shoulder arthroplasty (purple), and reverse shoulder arthroplasty (grey) from 2006 to 2015.

**Figure 3.**
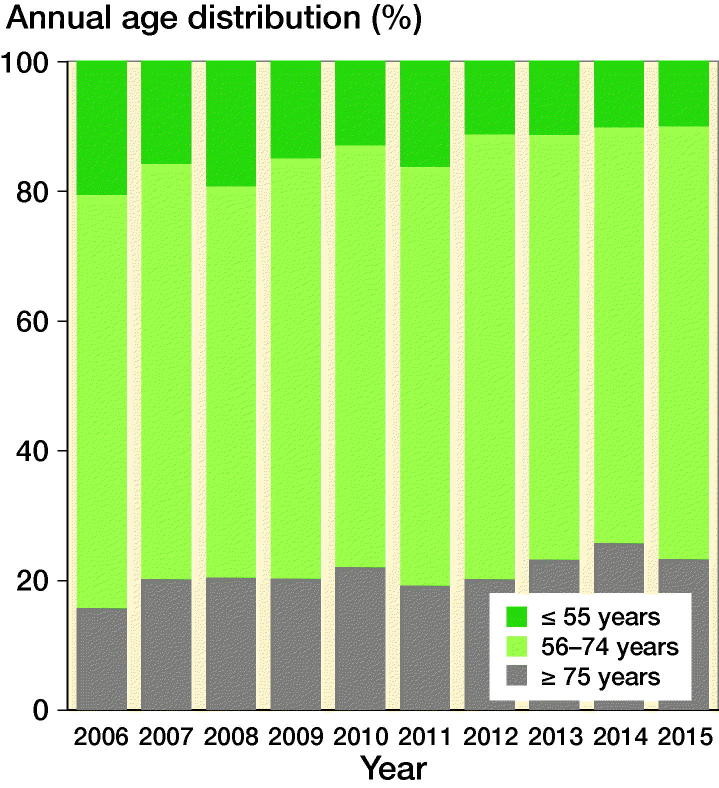
Proportion of patients who were 55 years or younger (dark green), between 56 and 74 years (light green), and 75 years or older (grey) from 2006 to 2015.

The number of shoulder arthroplasties for osteoarthritis increased from 141 in 2006 to 393 in 2015 (Figure 1). In the same period, the Danish population increased from 5,427,459 to 5,659,715 inhabitants. Thus, the number of arthroplasties increased by 179% whereas the population increased by 4%. 77% of all primary arthroplasties for osteoarthritis in 2006 were resurfacing hemiarthroplasties. Since then the proportion decreased to 3% in 2015. In the same period, the proportion of anatomical total shoulder arthroplasty and reverse shoulder arthroplasty increased from 3% to 53% and 7% to 27% respectively (Figure 2). The proportion of patients who were 55 years or younger decreased during the study period (Figure 3)

### Patient-reported outcome

51 (1.8%) patients died and 65 (2.3%) patients were revised within 1 year. Thus, the WOOS questionnaire was sent to 2,751 patients, of whom 69% returned a complete questionnaire. 6% of patients returned an incomplete questionnaire and 26% patients did not respond. The mean WOOS score was 59 (27) for patients who were 55 years or younger, 76 (24) for patients between 56 and 74 years, and 75 (25) for patients who were 75 years or older. The mean WOOS score was 73 (26) for women and 75 (24) for men. The mean WOOS score was 70 (26) after resurfacing hemiarthroplasties, 68 (26) after stemmed hemiarthroplasty, 82 (23) after anatomical total shoulder arthroplasties, and 74 (23) after reverse shoulder arthroplasties. In both the univariate and multivariable analysis, the outcome of anatomical total shoulder arthroplasty was better than that of any other arthroplasty type (Table 2).

**Table 2.** Linear regression model with difference, 95% confidence interval (CI), and with WOOS at 1 year as the dependent variable

Factor	Univariate model (95% CI)	Multivariable model (95% CI)
Year of operation (change per year)	1.8 (1.2 to 2.2)	1.0 (0.5 to 1.5)
Arthroplasty type **^a^**		
TSA	Reference	Reference
RHA	–12 (–9.4 to –15)	–6.6 (–3.5 to –9.8)
SHA	–14 (–10 to –17)	–11 (–7.8 to –15)
RSA	–7.2 (–2.5 to –12)	–7.0 (–2.4 to –12)
Sex		
Women	Reference	Reference
Men	2.4 (0.1–4.8)	5.6 (3.3 to 8.0)
Age		
≥≥ 75	Reference	Reference
56–74	1.1 (–1.7 to 3.9)	0.6 (–3.5 to 2.2)
≤≤ 55	–16 (–12 to –20)	–18 (–13 to –23)

**^a^**For abbreviations, see Table 1

In the univariate analysis the overall WOOS increased by a mean of 1.8 (CI 1.2–2.2) each year or 18 (CI 12–22) from 2006 to 2015 (Table 1). To investigate whether changes in demographics had influenced the change in WOOS from 2006 to 2015 we included sex and age category in a multivariable analysis where the overall WOOS increased by a mean of 17 (CI 13–21). This indicates that changes in demographics did not influence the overall WOOS score during the studied period.

We looked for interaction between year of surgery and arthroplasty type and found improved WOOS scores for anatomical total shoulder arthroplasty (mean change: 14, CI 5–23), and resurfacing hemiarthroplasty (mean change: 12, CI 5–19), but not for reverse shoulder arthroplasty (mean change: 11, CI –7–29) and stemmed hemiarthroplasty (mean change: –2.3, CI –14–10). Sex and age category were included in the model.

In a multivariable analysis, which included sex, age category, year of surgery, and arthroplasty type, the overall WOOS increased by a mean of 1.0 (CI 0.5–1.5) each year or 10 (CI 5–15) during the study period (Table 1). This indicate that the improvement in WOOS was influenced by changes in the distribution of arthroplasty types.

## Discussion

In this nationwide cohort study of patients with shoulder arthroplasty for osteoarthritis we found improved patient-reported outcome from 2006 to 2015. During the same period, there was an increased use particularly of anatomical total shoulder arthroplasty, but also of reverse shoulder arthroplasty. The outcome of anatomical total shoulder arthroplasty was superior to any other arthroplasty type and increased during the study period.

### Changes in the use of arthroplasty types

Information concerning the outcome of stemmed hemiarthroplasty and total shoulder arthroplasty was sparse until 2006. The first randomized trial was published in 2000 and included 27 total shoulder arthroplasties and 24 stemmed hemiarthroplasties, with mean ASES scores of 77 and 65 (Gartsman et al. 2000), respectively. Another randomized trial, which was published in 2005, included 20 total shoulder arthroplasties and 21 stemmed hemiarthroplasties. The mean WOOS score was 91 after total shoulder arthroplasty and 82 after hemiarthroplasty (Lo et al. 2005). Both studies were underpowered, and the differences were not statistically significant. A large observational study published in 2003 included 601 total shoulder arthroplasties and 89 stemmed hemiarthroplasties. With a mean follow-up of 3.5 years the mean Constant scores were 70 and 64, respectively. The difference was not statistically significant (Edwards et al. 2003). At that time, surgeons had concerns about rotator cuff problems and glenoid loosening after total shoulder arthroplasty (Bohsali et al. 2006). Thus, the inconclusive outcomes and the risk of glenoid loosening may explain why there were less than 20 total shoulder arthroplasties reported to the DSR each year in the beginning of the study period.

In 2004, Levy and Copeland (2004) published their results of resurfacing arthroplasty for osteoarthritis. They included 39 total resurfacing arthroplasties and 30 resurfacing hemiarthroplasties. The postoperative Constant scores were 62 and 58, respectively. To avoid late complications with glenoid loosening the authors recommended the resurfacing hemiarthroplasty for osteoarthritis except in patients with nonconcentric or saddle-shaped erosion of the glenoid. They concluded that the results were at least equal to those of stemmed shoulder arthroplasty and that the resurfacing arthroplasty had the advantage of a bone-preserving design, short operation time, and an easy revision, should the need for revision arthroplasty arise. A systematic review from 2009 supported their results and concluded that resurfacing hemiarthroplasty is a viable option for shoulder replacement, especially in young patients (Burgess et al. 2009). It is, of course, speculative but these studies are probably the main reason for the high proportion of resurfacing hemiarthroplasties in the first half of our study period.

The results of arthroplasty types were published in the annual reports from the DSR, presented at the annual meeting of the Danish Society for Shoulder and Elbow surgery in 2011, and later published. The results showed unpredictable patient-reported outcomes of the resurfacing hemiarthroplasty with a high proportion of disappointing results and a high rate of revision, especially in young patients (Rasmussen et al. 2014b), and the patient-reported outcome was inferior to that of total shoulder arthroplasty (Rasmussen et al. 2014a). Furthermore, data from the registry showed poor patient-reported outcomes of revision arthroplasty after failed resurfacing hemiarthroplasty, belying the hypothesis of an easy revision (Rasmussen et al. 2016).

A Cochrane review from 2010 included the 2 randomized trials by Gartsman et al. (2000) and Lo et al. (2005) with 88 arthroplasties and found a statistically significant superior ASES score after anatomical total shoulder arthroplasty compared with stemmed hemiarthroplasty (Singh et al. 2011). In 2011 the American Academy of Orthopedic Surgeons (AAOS) published its guidelines regarding the treatment of glenohumeral osteoarthritis and recommended anatomical total shoulder arthroplasty in patients with an intact rotator cuff (Izquierdo et al. 2011). Furthermore, in publications from 2012 and 2013 the results of the resurfacing hemiarthroplasty were questioned by the authors of small case series from independent centers (Al-Hadithy et al. 2012, Mechlenburg et al. 2013, Smith et al. 2013) and by data from the Norwegian Arthroplasty Register (Fevang et al. 2013).

The reason for the changed surgical practice in Denmark is speculative. It may be related to international trends for using total shoulder arthroplasty and reverse shoulder arthroplasty for osteoarthritis. This trend is, however, not supported by randomized trials or other strong evidence and we believed that data and publications from the DSR and from the national arthroplasty registries in Australia, Norway, Sweden, and New Zealand have contributed to the changed practice among Danish surgeons. Thus, our results indicate that a national registry can improve the outcome of shoulder arthroplasty by monitoring and reporting the outcome of arthroplasty types with inferior outcomes.

### Outcome of arthroplasty types

The results of arthroplasty types may not be directly comparable. The reverse shoulder arthroplasty has probably been used in patients with rotator cuff pathology or posterior subluxation. The excellent results of anatomical total shoulder arthroplasty might not have been the same if it had been used in the same patients. There are no existing randomized trials comparing the anatomical total shoulder arthroplasty and the reverse shoulder arthroplasty in patients with osteoarthritis and intact rotator cuff function. In our study, the outcome of anatomical total shoulder arthroplasty was better than reverse shoulder arthroplasty. This indicates that the anatomical total shoulder arthroplasty should be preferred in patients with an intact rotator cuff and no posterior subluxation, but the findings need to be confirmed by a randomized trial.

The results of the anatomical total shoulder arthroplasty were superior to stemmed hemiarthroplasty and resurfacing hemiarthroplasty. This support the guidelines from the AAOS (Izquierdo et al. 2011) recommending anatomical total shoulder arthroplasty in patients with glenohumeral osteoarthritis and intact rotator cuff function. However, it is important to stress the risk of selection bias.

Based on the existing literature (Izquierdo et al. 2011) and our results we consider the anatomical total shoulder arthroplasty as the gold standard in patients with an intact and well-functioning rotator cuff and without severe posterior glenoid wear. The reverse shoulder arthroplasty is reserved for patients with symptomatic rotator cuff or posterior subluxation, and the resurfacing hemiarthroplasty should be avoided. The stemmed hemiarthroplasty can be indicated in cases with severe glenoid bone loss where fixation of a glenoid component is not possible.

### Improved outcome from 2006 to 2015

Very few national arthroplasty registries have collected patient-reported outcome over a long period of time. So, to our knowledge this is the first study to report nationwide changes in patient-reported outcome after shoulder arthroplasty. We found a statistically and clinically significant improvement in WOOS of 18 from 2006 to 2015. The improvement was related to changes in the distribution of arthroplasty types during the studied period with a higher proportion in particular of anatomical total shoulder arthroplasty towards the end of the studied period. Changes in the distribution of sex and age group during the studied period had little influence on the improvement. We also found improved WOOS scores for anatomical total shoulder arthroplasty and resurfacing hemiarthroplasty during the studied period. Thus, the improvement in WOOS from 2006 to 2015 was primarily related to changes in the distribution of arthroplasty types and improved outcome of anatomical total shoulder arthroplasty and resurfacing hemiarthroplasty.

The reason for the improved outcome of anatomical total shoulder arthroplasty is speculative and cannot be deducted from our study. The reason for the improved outcome of resurfacing hemiarthroplasty is most likely related to the increased use of reverse shoulder arthroplasty and focus on the preoperative rotator cuff function. Better treatment selection, where the reverse shoulder arthroplasty is used in patients with rotator cuff insufficiency, has properly reduced the number of resurfacing hemiarthroplasties that fail because of rotator cuff insufficiency, and thereby improved the outcome of the few resurfacing hemiarthroplasties that were used towards the end of the studied period. It is important to stress that there might have been other reasons for the improved outcome which are not accounted for in the analysis.

### Limitations

The indications for surgery and for a specific arthroplasty type were not clearly defined. Thus, the risk of selection bias is important to keep in mind when the results are interpreted. Differences in preoperative WOOS scores between the arthroplasty types or between the year of operation might have influenced the comparison at 1 year. Not all patients returned a complete WOOS questionnaire, and any systematic differences in demographics or WOOS score between responders and non-responders would have influenced the results and interpretation. Finally, incorrect reporting may diminish the accuracy and reliability of the data.

## Conclusions

The patient-reported outcome of shoulder arthroplasty for osteoarthritis improved from 2006 to 2015. This may be related to different factors: improved outcome of anatomical total shoulder arthroplasty; the increased use of total shoulder arthroplasty towards the end of the study period; and better treatment selection, including the use of reverse shoulder arthroplasty in patients with poor rotator cuff function. The reason for the increased use of total shoulder arthroplasty is unknown but may be related to surgeons’ awareness of clinical results through annual reports from the DSR and other national arthroplasty registries. We recommend continued nationwide surveillance regarding the use of shoulder arthroplasty types.

All authors participated in the conception and design of the study, and in interpretation of the results. AA, SLJ, and JJ collected data. JVR and TWK performed the statistical analysis. AKS, SLJ, BSO, and JVR participated in the preparation of the manuscript.

The authors would like to thank the orthopedic surgeons in Denmark for data reporting. 

*Acta* thanks Randi Hole and Richard Page for help with peer review of this study.
